# Serum metabolomics analysis of biomarkers and metabolic pathways in patients with colorectal cancer associated with spleen-deficiency and qi-stagnation syndrome or damp-heat syndrome: a prospective cohort study

**DOI:** 10.3389/fonc.2023.1190706

**Published:** 2023-09-12

**Authors:** Min Zou, Yan-Sheng Zhang, Jin-Kai Feng, Hao Tu, Ming-Bin Gui, Ya-Nan Wang, Zi-Jie Yang, Zeng-Qiang Yang, Ming Xu, Wei-Qiang Wu, Feng Gao

**Affiliations:** ^1^ Department of Colorectal and Anal Surgery, The 940th Hospital of Joint Logistics Support Force of Chinese People’s Liberation Army, Lanzhou, China; ^2^ Department of Obstetrics and Gynecology, Gansu Provincial Maternity and Child-Care Hospital, Lanzhou, China; ^3^ Department of Hepatic Surgery VI, The Third Affiliated Hospital of Naval Medical University (Eastern Hepatobiliary Surgery Hospital), Shanghai, China; ^4^ Department of Colorectal Surgery, Chongqing Qijiang District People’s Hospital, Chongqing, China; ^5^ Department of Colorectal Surgery, Gansu Provincial Central Hospital, Lanzhou, China

**Keywords:** colorectal cancer (CRC), metabolomics, liquid chromatography-mass spectrometry (LC-MS), damp-heat syndrome (DHS), spleen-deficiency and qi-stagnation syndrome (SDQSS)

## Abstract

**Objective:**

To profile the serum metabolites and metabolic pathways in colorectal cancer (CRC) patients associated with spleen-deficiency and qi-stagnation syndrome (SDQSS) or damp-heat syndrome (DHS).

**Methods:**

From May 2020 to January 2021, CRC patients diagnosed with traditional Chinese medicine (TCM) syndromes of SDQSS or DHS were enrolled. The clinicopathological data of the SDQSS and DHS groups were compared. The serum samples were analyzed by liquid chromatography-mass spectrometry (LC-MS). The variable importance in the projection >1, fold change ≥3 or ≤0.333, and *P* value ≤0.05 were used to identify differential metabolites between the two groups. Furthermore, areas under the receiver operating characteristic (ROC) curve > 0.9 were applied to select biomarkers with good predictive performance. The enrichment metabolic pathways were searched through the database of Kyoto Encyclopedia of Genes and Genomes.

**Results:**

60 CRC patients were included (30 SDQSS and 30 DHS). The level of alanine aminotransferase was marginally significantly higher in the DHS group than the SDQSS group (*P* = 0.051). The other baseline clinicopathological characteristics were all comparable between the two groups. 23 differential serum metabolites were identified, among which 16 were significantly up-regulated and 7 were significantly down-regulated in the SDQSS group compared with the DHS group. ROC curve analysis showed that (S)-3-methyl-2-oxopentanoic acid, neocembrene, 1-aminocyclopropanecarboxylic acid, 3-methyl-3-hydroxypentanedioate, and nicotine were symbolic differential metabolites with higher predictive power. The top five enrichment signalling pathways were valine, leucine and isoleucine biosynthesis; lysosome; nicotine addiction; fructose and mannose metabolism; and pertussis.

**Conclusion:**

Our study identifies the differential metabolites and characteristic metabolic pathways among CRC patients with SDQSS or DHS, offering the possibility of accurate and objective syndrome differentiation and TCM treatment for CRC patients.

## Introduction

Colorectal cancer (CRC) is one of the most common malignant tumors, with the third highest incidence and the second highest mortality rates in the world (1). According to *Cancer Statistics in China, 2015* (2), the age-standardized morbidity and mortality rates of CRC in China were 17.81/100 000 and 8.12/100 000, ranking fourth and fifth, respectively. At present, the treatment options of CRC mainly include surgical resection, systemic chemotherapy, molecular targeted therapy, and immunotherapy. Radiation therapy with or without chemotherapy is used to treat rectal cancer (3). Due to the advance of diagnostic and treatment techniques of CRC, the long-term prognosis and quality of life of these patients are greatly improved. It has been reported that traditional Chinese medicine (TCM) treatment can inhibit tumor metastasis and growth (4, 5), accelerate postoperative rehabilitation (6), reduce postoperative complications (7), and decrease the side effects of chemotherapy in malignancies (8).

TCM is a unique medical theoretical system in China, and its therapeutic effect has been proved in clinical practice. Syndrome differentiation is the characteristic and foundation of disease diagnosis and treatment in TCM. *The Diagnosis and Treatment Guideline of Malignant Tumors Using TCM* issued by the Chinese Society of Traditional Chinese Medicine (2008 Edition) classifies TCM syndromes as 6 subtypes (9): spleen-deficiency and qi-stagnation syndrome (SDQSS), blood stasis and poison obstruction syndrome (SPOS), damp-heat syndrome (DHS), qi and blood deficiency syndrome (QBDS), spleen and kidney yang deficiency syndrome (SKYDS) and liver and kidney yin deficiency syndrome (LKYDS). Different TCM syndromes reflect various pathological features and stages of a certain disease. Conventional syndrome differentiation is mainly depended on the subjective judgment of the attending TCM physician, lacking the support of objective indicators.

Metabolomics is an integral part of systemic biology, which is a method of quantitative analysis of all metabolites in organisms and the relative relationship between metabolites and pathophysiological changes (10). In recent years, metabolomics plays an increasingly important role in TCM syndrome differentiation of various diseases, which shows promising value in the investigation of biological essence of TCM syndromes (11). Metabolomics can be used to identify symbolic metabolic biomarkers distinctive of different TCM syndromes. Nowadays, the commonly used analytical platforms of metabolomics are comprised of nuclear magnetic resonance (NMR), mass spectrometry, high performance liquid chromatography (HPLC) and their coupling technologies, such as liquid chromatography-mass spectrometry (LC-MS), gas chromatography-mass spectrometry (GC-MS) (12, 13).

In this study, LC-MS was used to detect the serum metabolic components of CRC patients with TCM syndromes of SDQSS or DHS. We analyzed the differential metabolites of CRC patients with SDQSS or DHS by multivariate statistical analysis and receiver operating characteristic (ROC) curve analysis, and identified the significant enrichment metabolic pathways. This study provides an objective reference for syndrome differentiation and TCM treatment of CRC.

## Materials and methods

### Ethical statement

This prospective cohort study was conducted in according to the ethical guidelines of Declaration of Helsinki (as revised in 2013). This study was approved by the Medical Ethics Committee of The 940th Hospital of Joint Logistics Support Force of Chinese People’s Liberation Army (approval number: 2020KYLL075). Individual written informed consent was obtained from all patients. Patients’ personal information have been anonymized to protect the privacy of patients.

### Patients

Patients with pathologically diagnosed CRC who were admitted to the 940th Hospital of Joint Logistics Support Force of Chinese People’s Liberation Army from May 2020 to January 2021 were consecutively enrolled. These patients were divided into the SDQSS and DHS groups according to TCM syndrome differentiation. Patients’ fasting peripheral venous blood was collected early in the morning and the serum was isolated and purified by centrifugation (1500 g, 10min, and 25°C) within 2h and stored at -80°C.

### Diagnostic criteria

The diagnostic criteria of CRC referred to the Chinese Colorectal Cancer Diagnosis and Treatment Guidelines (2020 edition) revised by the Chinese Society of Oncology (3). The TCM syndrome differentiation referred to the TCM Cancer Diagnosis and Treatment Guidelines (2008 edition) issued by the Chinese Association of TCM (9). The TCM syndrome of the patients was independently evaluated by two senior experts of the Department of TCM from our hospital. If the results were consistent, the TCM syndrome could be determined; otherwise, another physician participated in the differentiation until the correct TCM syndrome was obtained.

### Inclusion and exclusion criteria

The inclusion criteria included: (I) histopathologically diagnosed primary CRC; (II) age between 18 and 75 years; (III) patients’ TCM syndrome classified as SDQSS or DHS; (IV) patients did not receive preoperative neoadjuvant chemotherapy or radiotherapy; and (V) patients had sufficient vital organ functions.

The exclusion criteria included: (I) patients with active infectious diseases, such as tuberculosis; (II) patients who had immunodeficiency diseases, such as AIDS; (III) cases with other benign colorectal diseases or those without pathological diagnosis of CRC; (IV) complete clinical data were not available; and (V) patients who were incapable to cooperate for syndrome differentiation.

### CRC serum sample preparation

400 µL of cold methanol was added into 100 µL of serum samples and then vortex mixed for 60s. The mixture was then centrifuged at 12000 rpm for 10min at 4°C. All supernatant from each sample was transferred and dried in vacuum. Then the supernatant was dissolved with 150 µL of 2-chlorobenzalanine and 80% methanol mixed solution; and was filtered with 0.22 µm membrane to obtain the prepared samples for LC-MS. 20 µL of each sample was mixed into QC samples to correct for systematic errors caused by the analytical instrument (14–17). The remaining samples were subjected to LC-MS detection (Panomix, Suzhou, China).

### Data processing and multivariate data analysis

The LC-MS data were processed using the Proteowizard software (version 3.0.8789) and the XCMS package from R (version 3.6.3). Multivariate data analysis was achieved on the normalized LC-MS datasets with software package SIMCA-P (version 13.0) and the R language ropls package. Principal component analysis (PCA), partial least squares discriminant analysis (PLS-DA) and orthogonal partial least squares discriminant analysis (OPLS-DA) models were constructed to overview the distribution of different samples. Variable importance in the projection (VIP) value >1, fold change (FC) value ≥3 or ≤0.333, and *P* value ≤0.05 (18) were combined used to identify the differential metabolites between the two groups. Furthermore, an area under the ROC curve (AUC) > 0.9 was applied to select symbolic metabolic biomarkers with good predictive performance. Metabolite set enrichment analysis (MSEA) was performed by using online software MetaboAnalyst 4.0 and the Kyoto Encyclopedia of Genes and Genomes (KEGG) database.

### Statistical analysis

For continuous clinical data with normal distributions, means with standard deviation (SD) were shown, and the student’s *t* test was used to compare the differences. For skewed distributed continuous variables, medians with interquartile range (IQR) were expressed, and Mann-Whitney *U* test was used to compare the differences. Categorical data were exhibited as numbers and percentages, and compared using chi-square test or Fisher’s exact test as appropriate. Statistical significance was set as a *P* value less than 0.05 (two-tailed). SPSS version 24.0 software (SPSS Inc., Chicago, IL) was used for statistical analysis.

## Results

### Baseline clinicopathological characteristics of the SDQSS and DHS groups

As shown in **Supplementary Figure 1**, a total of 60 CRC patients with qualified serum samples, including 30 patients in the SDQSS group and 30 patients in the DHS group, were enrolled in this study. The baseline clinicopathological characteristics of the SDQSS and DHS groups of CRC patients are shown in **Table 1**, including sex, age, body mass index (BMI), primary site of CRC, tumor differentiation degree and pathological stage, carcinoembryonic antigen (CEA), carbohydrate antigen 19-9 (CA199), hemoglobin (HGB), red blood cell (RBC), white blood cell (WBC), platelet counts, alanine aminotransferase (ALT), aspartate aminotransaminase (AST), albumin (ALB), total bilirubin (TBil), blood urea nitrogen (BUN), and serum creatinine (Scr). The level of ALT was marginally significantly higher in the DHS group than the SDQSS group (*P* = 0.051). The other baseline clinicopathological characteristics were all comparable between the two groups.

**Table 1:** Clinicopathological characteristics of CRC patients with SDQSS or DHS.

Characteristics	SDQSS (n=30)	DHS (n=30)	*P* value
Sex			0.052
Male	17 (56.6%)	24 (80.0%)	
Female	13 (43.3%)	6 (20.0%)	
Age, years			0.196
≤ 65	12 (40.0%)	17 (56.7%)	
> 65	18 (60.0%)	13 (43.3%)	
BMI, kg/m^2^			0.271
≤ 24	18 (60.0%)	16 (53.3%)	
> 24	12 (40.0%)	14 (46.7%)	
Primary site			0.592
Colon	12 (40.0%)	10 (33.3%)	
Rectum	18 (60.0%)	20 (66.7%)	
Differentiation degree			0.100
Low	2 (6.7%)	8 (26.7%)	
Middle	26 (86.7%)	21 (70.0%)	
High	2 (6.7%)	1 (3.3%)	
pathological stage			0.073
I	1 (3.3%)	4 (13.3%)	
II	16 (53.3%)	8 (26.7%)	
III	9 (30.0%)	16 (53.3%)	
IV	4 (13.3%)	2 (6.7%)	
CEA, ng/ml			0.371
≤ 5	24 (80.0%)	21 (70.0%)	
>5	6 (20.0%)	9 (30.0%)	
CA19-9, U/ml			1.000
≤ 37	26 (86.7%)	26 (86.7%)	
>37	4 (13.3%)	4 (13.3%)	
HGB, g/L	135.5 (120.0–140.0)	137.0 (110.5–154.3)	0.589
RBC, 10^12^/L	4.45 ± 0.34	4.41 ± 0.59	0.744
WBC, 10^9^/L	5.89 ± 1.72	6.34 ± 2.11	0.369
PLT, 10^9^/L	195.6 ± 69.9	207.6 ± 74.8	0.522
ALT, U/L	12.6 ± 6.1	16.5 ± 8.8	0.051
AST, U/L	14.0 (12.75–17.00)	16.0 (13.00–18.25)	0.229
ALB, g/L	38.3 ± 2.8	38.7 ± 3.7	0.667
TBil, umol/L	9.0 (6.60–12.63)	10.7 (7.15–14.33)	0.773
BUN, mmol/L	5.1 (4.18–6.65)	5.8 (4.38–7.45)	0.549
Scr, umol/L	69.5 (62.75–73.0)	72.5 (64.75–84.75)	0.124

CRC, colorectal cancer; DHS, damp-heat syndrome; SDQSS, spleen deficiency and Qi stagnation syndrome; BMI, body mass index; CEA, carcinoembryonic antigen; CA19-9, carbohydrate antigen 19-9; HGB, hemoglobin; RBC, red blood cell; WBC, white blood cell; PLT, platelet; ALT, alanine aminotransferase; AST, aspartate aminotransferase; ALB, albumin; TBil, total bilirubin; BUN, blood urea nitrogen; Scr, serum creatinine.

### Quality control (QC)

Theoretically, all QC samples were identical, but systematic errors in the process of sample extraction, detection and analysis were unavoidable, which would lead to potential differences among QC samples. As shown in **Supplementary Figure 2**, the QC samples on PCA score plots of positive and negative ion modes were clustered with good repeatability, which indicated that the data were reliable and the database building system was stable.

### PCA and PLS-DA analysis of metabolomics profiles in the SDQSS and DHS groups of CRC patients

**Figure 1:**
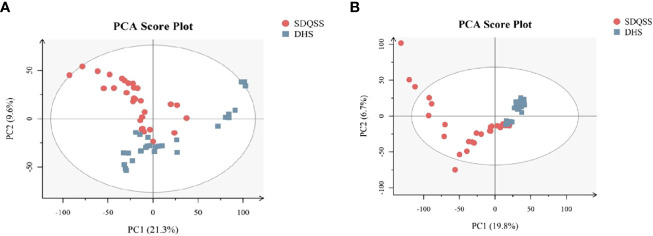
PCA score plots of the SDQSS and DHS groups. **(A)** PCA in positive ion mode; **(B)** PCA in negative ion mode. PCA, principal component analysis; SDQSS, spleen-deficiency and qi-stagnation syndrome; DHS, damp-heat syndrome.

Principal component analysis (PCA) reflects the original state of metabolomic data. The aggregation and dispersion degree of samples can be observed from PCA score plots. As shown in **Figure 1**, the spatial distribution of principal components in metabolic spectra of SDQSS and DHS was discrete. The results demonstrated that there were obvious differences in serum metabolites between the two TCM syndrome groups.

**Figure 2:**
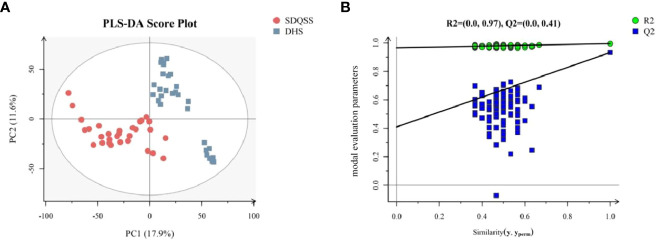
**(A)** PLS-DA score plots of the SDQSS and DHS groups; **(B)** PLS-DA permutation plot. In **Figure 2B**, the X-axis represents the similarity between the real grouping of samples and 100 random grouping, and the Y-axis indicates the model evaluation parameters. The cross-validation of PLS-DA permutation model mainly refers to parameters such as R2X, R2Y, and Q2. R2X is the interpretability of model X variable (independent variable), R2Y is the interpretability of model Y variable (dependent variable), and Q2 is the predictability of the model. Points R2 and Q2 in the upper right corner of **Figure 2B** represent the model parameters of real grouping. Usually, it is better when R2 and Q2 are both larger than 0.5, and the maximum values of R2 and Q2 are 1. When the R2 value is small, it means that the repeatability in the test set is poor (the background noise is high); when the Q2 value is small, it indicates that there is high background noise in the test set, or the model has more outlier. Permutation plot can help to effectively evaluate whether the PLS-DA model is over-fitted. The judging criteria are one of the follows: ① all blue Q2 points are lower than the rightmost original blue Q2 point from left to right; ② the intersection of the regression line at Q2 in the Y-axis is less than or equal to 0. PLS-DA, partial least squares discriminate analysis; SDQSS, spleen-deficiency and qi-stagnation syndrome; DHS, damp-heat syndrome.

PLS-DA can specify and group the samples during analysis, and can discriminate the differences in various samples more sensitively. As shown in PLS-DA score plot (**Figure 2A**), a significant separation of dots in different colors was observed, which also indicated significant differences existed in serum metabolic spectrum between the two groups.

In order to confirm there was no overfitting in the PLS-DA model, a permutation test was conducted. As shown in **Figure 2B**, all blue Q2 points from the leftmost were lower than the rightmost original blue Q2 point, indicating that there was no overfitting in the PLS-DA model, and it could be used to identify the differentially expressed metabolites and the related metabolic pathways.

### Identification of differential metabolites among the SDQSS and DHS samples

**Figure 3:**
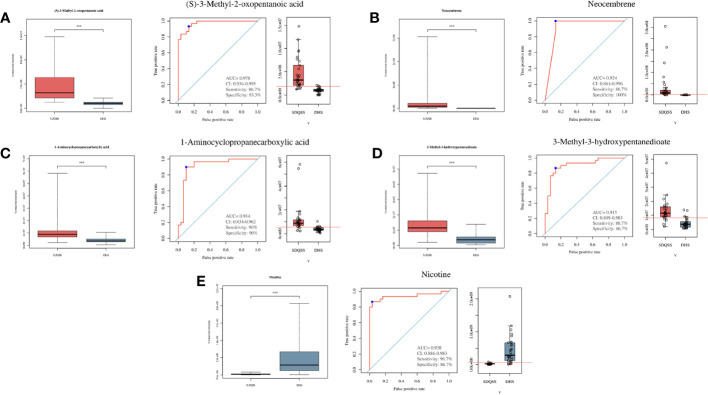
Box plots and ROC curves of 5 selected differential metabolites with AUC > 0.9. **(A)** (S)-3-methyl-2-oxopentanoic acid; **(B)** neocembrene; **(C)** 1-aminocyclopropanecarboxylic acid; **(D)** 3-methyl-3-hydroxypentanedioate; **(E)** nicotine. ROC, receiver operating characteristic; AUC, the area under the ROC curve; CI, confidence interval; SDQSS, spleen-deficiency and qi-stagnation syndrome; DHS, damp-heat syndrome.

A total of 3309 metabolites were identified by LC-MS analysis, of which 2775 were up-regulated and 534 were down-regulated. According to the selection criteria, 23 differential metabolites were screened out, among which 16 were significantly up-regulated and 7 were significantly down-regulated in the SDQSS group compared with the DHS group (**Table 2**). In addition, 5 differential metabolites with high predictive accuracy and diagnostic power were selected (AUC>0.9), including (S)-3-methyl-2-oxopentanoic acid, neocembrene, 1-aminocyclopropanecarboxylic acid, 3-methyl-3-hydroxypentanedioate, and nicotine. Among them, nicotine is the landmark metabolite of the DHS group, and (S)-3-methyl-2-oxopentanoic acid, neocembrene, 1-aminocyclopropanecarboxylic acid, 3-methyl-3-hydroxypentanedioate are the landmark metabolites of the SDQSS group. The box plots and ROC curves of these 5 metabolites are exhibited in **Figure 3**.

**Table 2:** Differential metabolites in the serum of CRC patients with SDQSS compared with those with DHS.

Metabolite	VIP	FC	*P* value	FDR	Regulation direction	AUC	CI1	CI2	specificity	sensitivity
(S)-3-Methyl-2-oxopentanoic acid	1.97	4.63	3.45E-10	5.28E-08	up	**0.978**	0.936	0.995	0.87	0.93
Neocembrene	1.15	437.66	1.65E-09	1.51E-07	up	**0.924**	0.861	0.996	0.87	1.00
1-Aminocyclopropanecarboxylic acid	1.42	3.15	4.31E-08	1.81E-06	up	**0.914**	0.834	0.962	0.90	0.90
3-Methyl-3-hydroxypentanedioate	1.95	3.01	8.35E-08	2.96E-06	up	**0.915**	0.809	0.983	0.87	0.87
Triacetate lactone	1.64	4.19	2.87E-06	4.60E-05	up	0.857	0.774	0.933	0.90	0.73
Erucic acid	1.10	3.89	5.86E-06	8.07E-05	up	0.851	0.769	0.936	0.87	0.73
5-Methyl-2-furancarboxaldehyde	1.53	3.88	9.51E-06	1.17E-04	up	0.846	0.726	0.908	0.67	0.80
6-Acetyl-D-glucose	1.42	3.64	8.56E-04	3.83E-03	up	0.76	0.58	0.855	0.67	0.73
Coniferyl alcohol	1.41	3.58	1.00E-03	4.34E-03	up	0.746	0.634	0.889	0.67	0.77
(S)-beta-Tyrosine	1.46	4.52	1.68E-03	6.41E-03	up	0.76	0.68	0.844	0.63	0.83
D-erythro-3-Methylmalate	1.03	3.30	1.77E-03	6.68E-03	up	0.747	0.605	0.851	0.67	0.77
4-Hydroxycinnamic acid	1.67	3.07	7.30E-04	3.37E-03	up	0.934	0.87	0.975	0.83	0.90
9-OxoODE	1.75	3.85	7.60E-07	9.95E-06	up	0.889	0.772	0.939	0.87	0.87
L-Aspartate-semialdehyde	1.61	3.61	2.00E-06	2.06E-05	up	0.859	0.74	0.966	0.77	0.87
5-Aminopentanoic acid	1.07	4.14	7.74E-06	6.06E-05	up	0.842	0.759	0.927	0.80	0.80
D-Sorbose	1.00	3.18	8.56E-04	2.86E-03	up	0.753	0.622	0.864	0.60	0.83
4-Pyridoxic acid	1.89	0.19	1.04E-04	7.40E-04	down	0.802	0.664	0.896	0.93	0.77
Mannitol 1-phosphate	1.52	0.15	2.68E-04	1.57E-03	down	0.758	0.593	0.88	0.97	0.73
Nicotinic acid	1.65	0.29	6.90E-04	3.24E-03	down	0.756	0.628	0.856	0.77	0.73
O-Acetylcarnitine	1.65	0.14	1.68E-03	6.41E-03	down	0.737	0.602	0.858	0.87	0.70
Nicotine	1.65	0.06	5.97E-09	3.11E-07	down	**0.938**	0.886	0.983	0.97	0.87
D-Mannose	1.46	0.28	2.18E-05	1.39E-04	down	0.826	0.741	0.914	0.83	0.73
Gluconolactone	1.29	0.27	2.01E-04	8.52E-04	down	0.783	0.629	0.867	0.93	0.67

CRC, colorectal cancer; DHS, damp-heat syndrome; SDQSS, spleen deficiency and Qi stagnation syndrome; VIP, variable importance in the projection; FC, fold change; FDR, false discovery rate; AUC: The area under receiver operating characteristic (ROC) curves; CI1: the lower limit of 95% confidence interval; CI2: the upper limit of 95% confidence interval.

AUC in bold denotes values > 0.9.

### Hierarchical clustering and metabolic pathways

**Figure 4:**
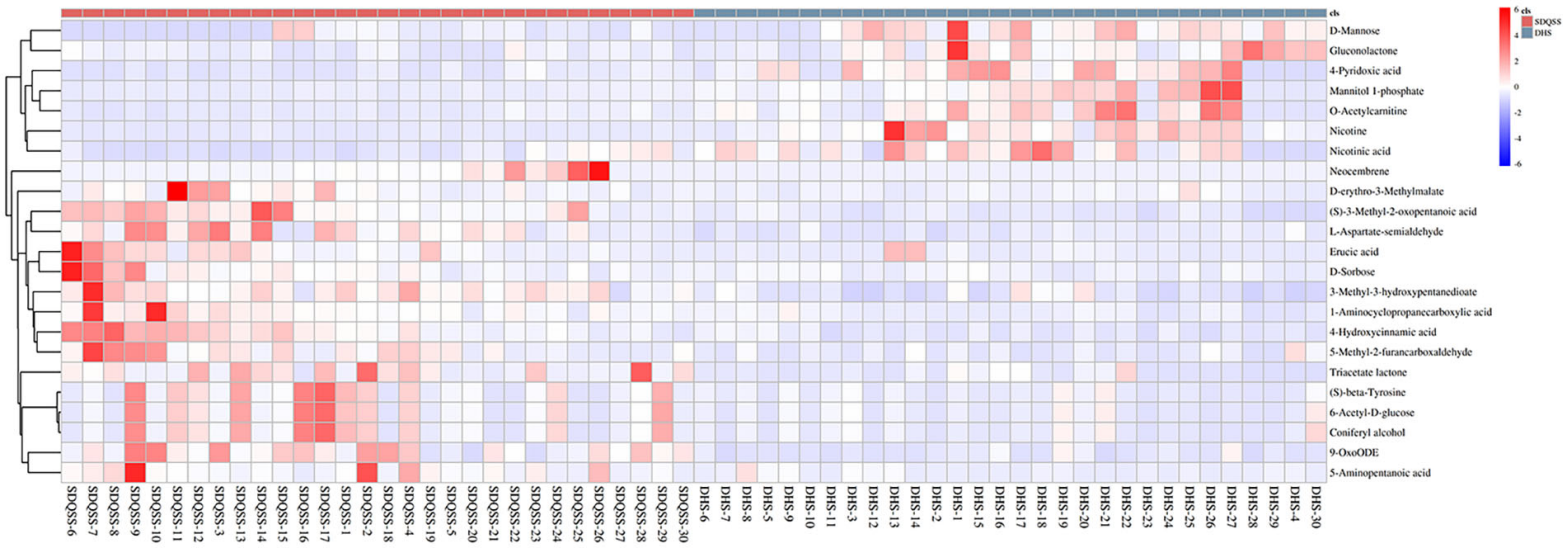
Hierarchical clustering heat map of differential metabolites between the SDQSS and DHS groups. SDQSS, spleen-deficiency and qi-stagnation syndrome; DHS, damp-heat syndrome.

Hierarchical clustering is commonly used for unsupervised clustering. It is performed when taking the relative contents of metabolites under different experimental conditions as metabolic levels. The results showed that CRC patients with SDQSS or DHS syndrome could be distinguished well (**Figure 4**).

**Figure 5:**
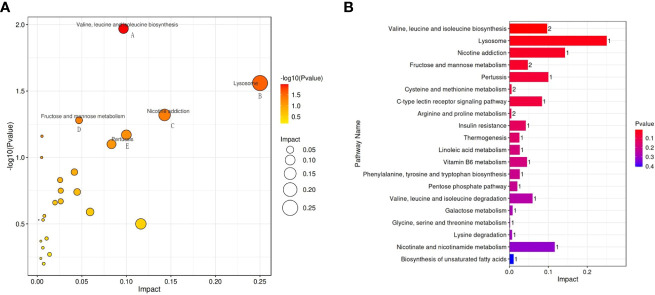
Bubble diagram **(A)** and bar chart **(B)** of enriched metabolic pathways of the SDQSS and DHS groups. SDQSS, spleen-deficiency and qi-stagnation syndrome; DHS, damp-heat syndrome.

The possible metabolic pathways pertaining to CRC with SDQSS or DHS were analyzed with MetaboAnalyst 4.0, a free online metabolomics analysis platform on the basis of high-throughput KEGG metabolic pathways database. The pathway impact value was calculated by pathway topology analysis. For SDQSS versus DHS, the top 5 potential enrichment signalling pathways were valine, leucine and isoleucine biosynthesis; lysosome; nicotine addiction; fructose and mannose metabolism; and pertussis (**Figure 5**).

## Discussion

CRC is one of the most common malignant tumors and its incidence and mortality rates are gradually increasing in the world (1). At present, the diagnosis and treatment of CRC have developed rapidly, and the prognosis of these patients has greatly improved. TCM is a unique and long recognized theoretical system in China. TCM has been widely used as part of adjuvant therapy and comprehensive treatment for malignancies in clinical practice. The advantages of integrating TCM and Western medicine are becoming increasingly obvious, such as relief of postoperative pain, accelerating postoperative rehabilitation, and reduction of chemotherapeutic side effects. Syndrome differentiation is the foundation of TCM treatment, and the accuracy of syndrome differentiation can be interfered because of the subjectivity of attending doctors. Thus, accurate differentiation of TCM syndromes based on objective materials and quantitative biomarkers is particularly important.

TCM holds that CRC is caused by a series of internal and external negative factors, such as deficiency of vital Qi, weakness of spleen and stomach, external evils invasion, anxiety and depression, or improper diet, all of which lead to endogenous dampness and heat, qi stagnation, blood stasis and toxin stagnation. DHS and SDQSS are two basic TCM syndromes of CRC. DHS is the characteristic TCM syndrome type of early stage of CRC, with clinical manifestations of abdominal distension, mucous bloody stool, red tongue, yellow and greasy fur, and slippery pulse. SDQSS belongs to the TCM syndrome type of relatively advanced stage of CRC. Clinically, it is mainly manifested as abdominal pain, anorexia, mental fatigue, sallow complexion, thin stool, pale tongue, thin and greasy fur, and thready pulse. There is great implications to discriminating these two TCM syndromes in clinical practice, because the TCM treatment approaches mainly depend on CRC patients’ syndrome types.

Recently, an increasing number of studies have applied metabolomics to distinguish TCM syndromes of different diseases. Jiang et al. (19) used nuclear magnetic resonance (NMR) to analyze the plasma metabolites of diabetic patients with kidney-yin deficiency syndrome (KYDS), and found that the levels of creatinine, citric acid, trimethylamine oxide, phenylalanine and tyrosine were decreased, whereas the levels of alanine, glycine and taurine were increased, which can be used as the landmark metabolites for the diagnosis of KYDS. Chen et al. (20) found that phlegm-dampness stasis syndrome (PDSS) was mainly attributed to the accumulation of harmful metabolites, while LKYDS was mainly caused by the lack of protective metabolites. The serum metabolic patterns in cellular oxidation, inflammatory reaction and energy metabolism of these two syndromes were significantly different.

In this study, the clinicopathological characteristics of CRC patients in the SDQSS and DHS groups were compared. A marginally significant increase of ALT was observed in the DHS group compared with the SDQSS group (*P* = 0.051). Next, we used LC-MS to analyze the metabolic profiles of serum samples from CRC patients with SDQSS or DHS TCM syndrome. In our study, 23 differential metabolites were identified in the two groups. ROC curve analysis of these differential metabolites showed that areas under the ROC curves (AUC) of (S)-3-methyl-2-oxopentanoic acid, neocembrene, 1-aminocyclopropanecarboxylic acid, 3-methyl-3-hydroxypentanedioate, and nicotine were larger than 0.9, which indicated these metabolites were sensitive and specific serum biomarkers to distinguish CRC patients with SDQSS from those with DHS.

The characteristic differential metabolite with the highest discrimination ability for CRC with DHS was nicotine, which was markedly upregulated in patients with DHS compared with those with SDQSS. It was reported that 4-(methylnitrosamine)-1-(3-pyridine)-1-butanone (NNK) derived from nicotine could promote the formation of cell spheres and increase the expression of cell surface markers CD44, OCT4, C-MYC and NANOG in HCT8 and DLD-1 cells (21), while exposure to NNK could significantly enhance the proliferation and growth ability of CRC cells. Nicotine could promote the growth and metastasis of CRC through downregulation of miR-200c (21); it could also stimulate the invasion and metastasis of colon cancer cells *in vitro* by activating the downstream signalling pathways of nAchRs and p38 MAPK (22, 23). Thus, the remarkable increase of serum nicotine level in CRC patients with DHS syndrome can reflect a high risk of postoperative recurrence and metastasis. Regular monitoring of serum nicotine level in CRC patients with DHS may assist early detection of tumor recurrence and metastasis in clinical practice.

The characteristic differential metabolite that was significantly upregulated in the SDQSS group was 4-hydroxycinnamic acid, an important polyphenol in the plant manganese-containing acid biosynthetic pathway, mainly found in cereals, fruits and vegetables (24). This compound has a range of beneficial pharmacological properties, including powerful antioxidant, anti-inflammatory, anti-ulcer (25), and anti-cancer effects (26). Neog et al. (27). found that the anti-inflammatory effects of hydroxycinnamic acid were mediated through inhibition of inflammation-related proteins including nuclear factor kappa B (NF-kB), inducible nitric oxide synthase (iNOS) and cyclooxygenase-2 (COX-2). Ko et al. (28) investigated the effects of 4-hydroxycinnamic acid on the inflammatory response in asthma using an allergic asthma mouse model. They found that 4-hydroxycinnamic acid reduced the levels of IL-5 and IL-13 in bronchoalveolar lavage fluid (BALF), alleviated airway inflammation and mucus overproduction induced by ovalbumin exposure. In addition, 4-hydroxycinnamic acid could inhibit the increased levels of NF-kB, iNOS and COX-2, and also reduced matrix metalloproteinase-9 (MMP-9) activity and protein levels (28). The elevated 4-hydroxycinnamic acid level may indicate a better anti-inflammatory effect in CRC patients with SDQSS compared to patients with DHS.

On the other hand, in this study, 5 classical metabolic pathways related to 23 discriminating metabolites were found in the SDQSS and DHS groups. These pathways suggest that severe metabolic disturbances occur during the development and progression of CRC with different TCM syndromes. The enrichment of these signalling pathways may correlate with these syndrome types.

Several limitations of our study should be acknowledged. First, the sample size of our study is insufficient. Only 30 samples were enrolled in each of the two groups, which is not large enough for the confirmative association between metabolic profiles and various TCM syndromes in CRC patients. With the sample size increased beyond 60 patients, the variables with marginally significant difference, such as ALT and pathological stage, may become statistically significantly different; hence, proper sample collection practices are needed to avoid confounding effects. Second, the detailed mechanisms underlying changes of metabolites in different CRC syndromes should be deciphered with the integration of transcriptomics and proteomics. Third, healthy participants were not included as baseline control. Last, there is a lack of metabolic profiling analysis of tumor tissues from CRC patients with different syndromes.

## Conclusion

Our study identifies the differential metabolites and characteristic metabolic pathways of CRC patients with SDQSS or DHS, TCM syndrome offering the possibility of accurate and objective syndrome differentiation and TCM treatment for CRC patients. Nevertheless, the results of this study need to be verified by further research.

## Data availability statement

The raw data supporting the conclusions of this article will be made available by the authors, without undue reservation.

## Ethics statement

The studies involving humans were approved by the Medical Ethics Committee of The 940th Hospital of Joint Logistics Support Force of Chinese People’s Liberation Army (approval number: 2020KYLL075). The studies were conducted in accordance with the local legislation and institutional requirements. The participants provided their written informed consent to participate in this study.

## Author contributions

Conceptualization and design: MZ and FG; Administrative support and funding acquisition: FG; Provision of study materials or patients: HT, M-BG, Y-NW, Z-JY, Z-QY, MX, and W-QW; Collection and assembly of data: MZ, HT, M-BG, Y-NW, and Z-JY; Data analysis and interpretation: MZ, Y-SZ, and J-KF; Manuscript writing and editing: MZ, Y-SZ, and J-KF. All authors contributed to the article and approved the submitted version.

## Supplementary material

Supplementary Figure 1 Study design and patients enrollment. DHS, damp-heat syndrome; SDQSS, spleen-deficiency and qi-stagnation syndrome.

Supplementary Figure 2 PCA score plots of QC samples. **(A)** PCA in positive ion mode; **(B)** PCA in negative ion mode. PCA, principal component analysis; QC, quality control.
