# Supplementary figures and images for: Serum metabolomics analysis of biomarkers and metabolic pathways in patients with colorectal cancer associated with spleen-deficiency and qi-stagnation syndrome or damp-heat syndrome: a prospective cohort study
Source: Front Oncol. 2023 Sep 12;13:1190706. doi: 10.3389/fonc.2023.1190706 (PMC10523394; doi:10.3389/fonc.2023.1190706)

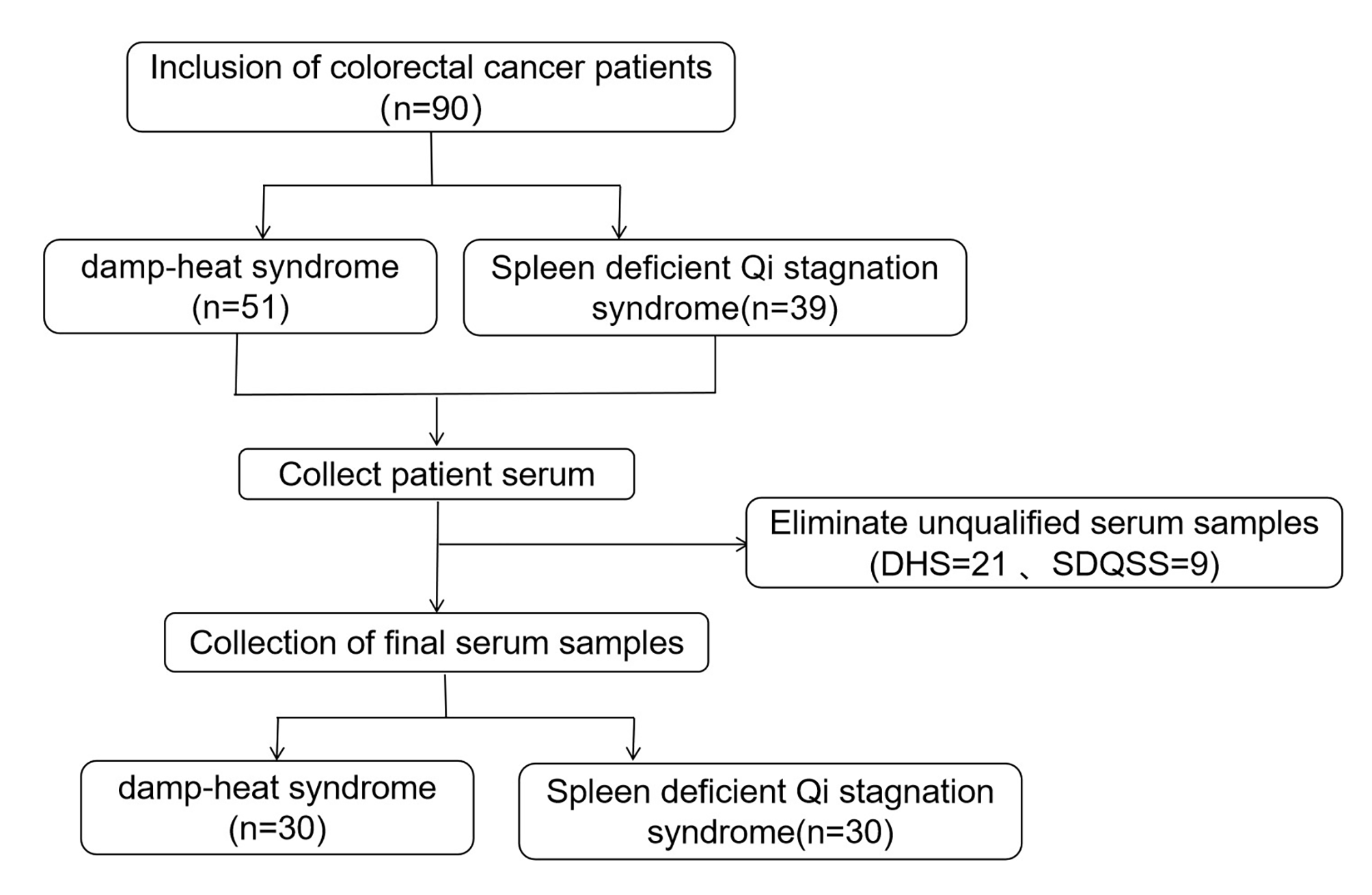

Supplement: Supplementary Figure 1 — Study design and patients enrollment. DHS, damp-heat syndrome; SDQSS, spleen-deficiency and qi-stagnation syndrome. [file Image_1.tif]

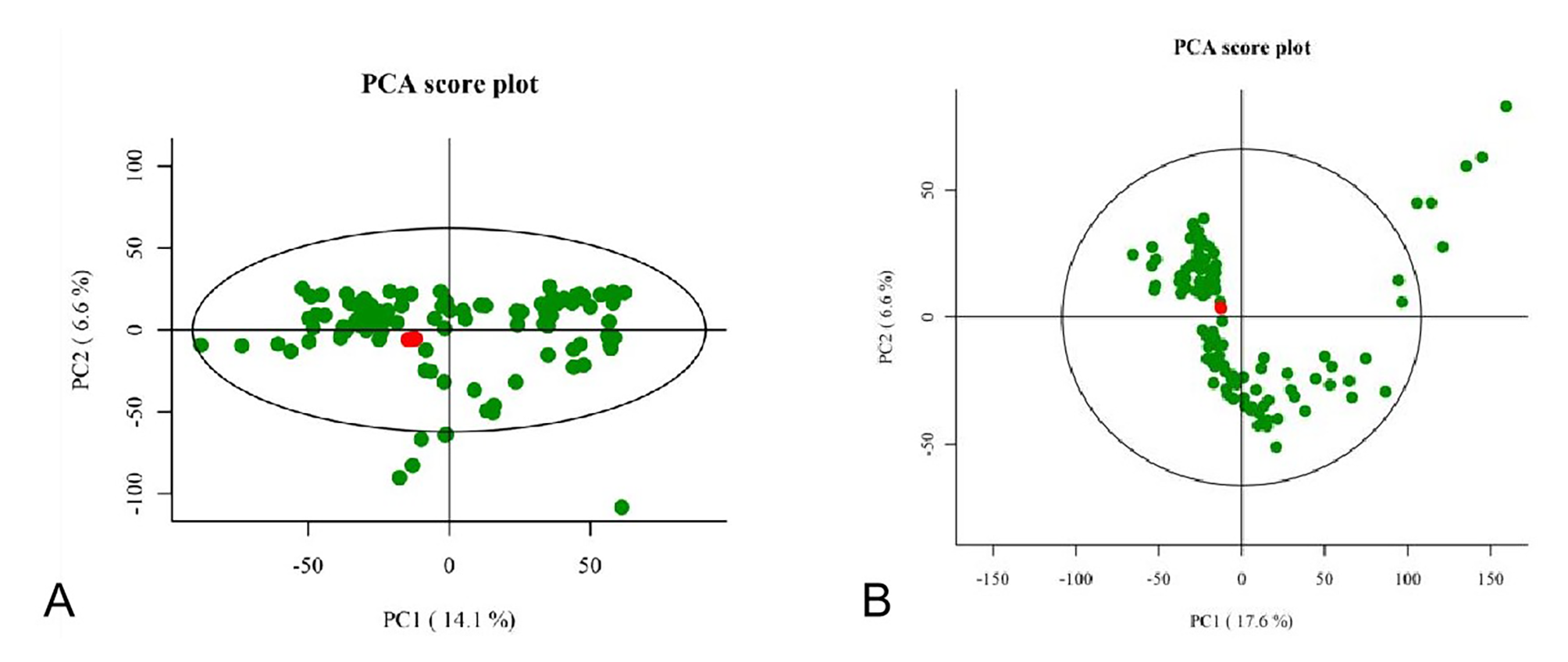

Supplement: Supplementary Figure 2 — PCA score plots of QC samples. (A) PCA in positive ion mode; (B) PCA in negative ion mode. PCA, principal component analysis; QC, quality control. [file Image_2.tif]
